# Hemodynamic biomarkers in symptomatic intracranial atherosclerotic stenosis: evidence boundaries and research pathways from prognostic stratification to treatment-effect prediction

**DOI:** 10.3389/fneur.2026.1980919

**Published:** 2026-10-15

**Authors:** Ruying Hu, Zizhao Zhang, Zipeng Wang, Jianmin Liu, Lei Zhang, Hongjian Zhang

**Affiliations:** 1 Oriental Pan-Vascular Devices Innovation College, University of Shanghai for Science and Technology, Shanghai, China; 2 Neurovascular Center, Changhai Hospital, Naval Medical University, Shanghai, China

**Keywords:** biomarkers, cerebral perfusion, computational fluid dynamics, Hemodynamics, symptomatic intracranial atherosclerotic stenosis

## Abstract

Symptomatic intracranial atherosclerotic stenosis (sICAS) has traditionally been stratified primarily according to angiographic stenosis severity. However, this anatomical approach does not adequately capture the heterogeneity of ischemic mechanisms, distal hemodynamic compromise, or treatment response, prompting increasing interest in hemodynamic biomarkers for mechanism-based risk stratification. In this review, we propose an evidence framework that evaluates these biomarkers across measurement validity, mechanistic coherence, prognostic association, treatment-effect prediction, external validation, and clinical utility, while integrating lesion function, distal perfusion, compensatory capacity, and parallel embolic and perforator pathways. Although several measures, particularly distal hypoperfusion and borderzone infarction, have shown prognostic value, the evidence identified in this review remains dominated by single-center studies, platform-specific definitions, and incompletely validated thresholds. Among the completed direct sICAS studies identified, we found no prospective validation of a hemodynamic biomarker as a treatment-effect modifier. Translation requires standardized measurement, prespecified biomarker definitions, and external validation. Claims of treatment-effect modification additionally require prespecified interaction analyses within appropriate treatment comparisons, preferably randomized. Biomarker-guided treatment selection should be supported by reliable estimates of absolute benefits and harms and evidence of clinical utility.

## 1 Introduction

Symptomatic intracranial atherosclerotic stenosis (sICAS) is an important cause of ischemic stroke (1–5). The Warfarin–Aspirin Symptomatic Intracranial Disease (WASID) study demonstrated that recent symptoms and severe stenosis of 70–99% were associated with an increased risk of ischemic stroke in the territory of the qualifying artery, establishing stenosis severity as an important prognostic factor (6, 7). However, lesions of similar angiographic severity may be associated with markedly different infarct patterns, collateral compensation, distal perfusion status, and mechanisms of recurrence. Thus, the degree of luminal stenosis alone does not fully account for the clinical and pathophysiological heterogeneity of sICAS (8–10).

Findings from randomized endovascular trials further indicate that severe anatomical stenosis alone is insufficient for treatment selection. In the Stenting and Aggressive Medical Management for Preventing Recurrent Stroke in Intracranial Stenosis (SAMMPRIS) trial, Wingspan stenting plus aggressive medical management was associated with a higher 30-day rate of stroke or death than aggressive medical management alone (14.7% vs. 5.8%), and long-term follow-up continued to support the medical-management strategy (11, 12). Similarly, the Vitesse Intracranial Stent Study for Ischemic Stroke Therapy (VISSIT) failed to demonstrate a clinical benefit of balloon-expandable stenting (13, 14). Even with more stringent patient-selection criteria and operator credentialing, the China Angioplasty and Stenting for Symptomatic Intracranial Severe Stenosis (CASSISS) trial did not show superiority of stenting plus medical therapy over medical therapy alone (14, 15). By contrast, the Balloon Angioplasty for Symptomatic Intracranial Artery Stenosis (BASIS) trial, a randomized open-label trial with blinded endpoint assessment conducted at 31 stroke centers in China, showed that submaximal balloon angioplasty plus aggressive medical management reduced the 1-year primary composite outcome compared with medical management alone in selected patients with 70–99% sICAS (4.4% vs. 13.5%; HR, 0.32; 95% CI, 0.16–0.63). The composite endpoint combined early safety events—stroke of any type or all-cause death within 30 days of enrollment or balloon angioplasty of the qualifying lesion—with later events: ischemic stroke in the qualifying artery territory or revascularization of that artery from day 31 through month 12 after enrollment (16). However, this benefit should be interpreted alongside procedural risk: 30-day stroke or death occurred in 3.2% versus 1.6%, whereas qualifying-territory ischemic stroke from day 31 to 1 year occurred in 0.4% versus 7.5% in the angioplasty and medical-management groups, respectively. Procedure-related complications occurred in 17.4% of patients undergoing angioplasty, most commonly arterial dissection. Because the trial was conducted in experienced Chinese centers with standardized operator training, its procedural safety and efficacy may not be directly generalizable to other settings (16). In this context, “submaximal” refers to the deliberate avoidance of complete vessel expansion. BASIS recommended a balloon inflation diameter corresponding to 50–70% of the proximal reference-vessel diameter to balance luminal gain against the risk of vascular injury (16). Current clinical management remains centered on aggressive medical therapy and comprehensive vascular risk-factor control (1, 17–22). Collectively, these trials indicate that treatment outcomes are jointly determined by the therapeutic approach, patient selection, and procedure-related risk (23, 24).

Accordingly, precision stratification in sICAS should extend beyond identifying patients at high risk of recurrence. When the average relative treatment effect and safety profile of an intervention are established, a higher baseline risk may translate into a greater absolute treatment benefit and thereby help determine whether a clinically meaningful treatment threshold has been reached. Prognostic risk alone, however, generally does not determine which strategy is preferable among balloon angioplasty, stenting, blood-pressure management, or intensified antithrombotic therapy. Such decisions require estimation of treatment-specific differences in clinical outcomes and net clinical benefit and therefore cannot be derived directly from recurrence risk. On this basis, the present review reorganizes the hemodynamic evidence by distinguishing measurement validity, mechanistic interpretation, prognostic association, treatment-effect prediction, and clinical utility, and proposes candidate biomarker combinations that can be prospectively tested, validated, or refuted.

Recently, Guo et al. systematically reviewed endovascular treatment for sICAS from the perspectives of patient selection, lesion imaging, interventional strategies, novel devices, and guideline and consensus recommendations, and proposed the integration of high-resolution MRI with functional assessment to improve precision in patient selection (25). A previous systematic review of computational fluid dynamics (CFD) studies summarized technical and clinical investigations of pressure-, velocity-, and shear-related hemodynamic metrics (26). Building on these studies, the present review focuses on the evidentiary role currently supported for each hemodynamic measure, evaluates whether individual measures can progress from recurrence-risk stratification to prediction of treatment-specific effects, and identifies the types of future studies required to test or refute these inferences.

## 2 Literature search and evidence evaluation

This narrative review was not conducted as a systematic review or meta-analysis. Searches were last updated on September 17, 2026, in PubMed, Web of Science Core Collection, and Scopus. Eligibility was restricted to English-language publications. Representative English-language search concepts combined (1) sICAS/ICAD terms (“intracranial atherosclerotic stenosis” OR “intracranial atherosclerotic disease” OR ICAS OR ICAD) with (2) hemodynamic terms (“pressure ratio” OR “fractional flow” OR Angio-FF OR “CT-FFR” OR “wall shear stress” OR perfusion OR Tmax OR “quantitative magnetic resonance angiography” OR collateral* OR “cerebrovascular reserve”) and, where relevant, (3) prediction terms (biomarker* OR interaction OR “heterogeneity of treatment effect”). The primary evidence window was 2016–2026, with priority to the most recent 5 years. We included human studies reporting measurement validity, mechanistic association, prognosis, treatment comparison, or registered trial design relevant to symptomatic intracranial stenosis; we excluded nonhuman work, studies without usable hemodynamic or clinical-outcome information, and duplicate reports. Landmark trials and related disease settings were retained only to contextualize treatment strategy, procedural harm, biomarker definition, or study design. “Representative” and “major” denote studies selected to cover each prespecified topic, with priority to randomized comparisons and prespecified interaction analyses, prospective multicenter cohorts, longitudinal sICAS cohorts, systematic reviews/meta-analyses, and technical validation studies. Direct same-cohort cross-level associations, cross-cohort synthesis, and physiologically plausible inferences were recorded separately. No protocol registration, dual screening, formal risk-of-bias assessment, or quantitative pooling was undertaken; absence claims are therefore limited to the evidence identified through this approach.

## 3 Conceptual and evaluation framework for hemodynamic biomarkers

### 3.1 Hemodynamic digital features and candidate biomarkers

An individual measurement of pressure ratio (PR), angiography-derived fractional flow (Angio-FF), wall shear stress (WSS), Tmax volume, or quantitative magnetic resonance angiography (QMRA) flow should initially be regarded as a digital feature. A digital feature may be investigated as a candidate biomarker when its measurement method and intended context of use are clearly specified and its proposed role is supported by a biological rationale or preliminary empirical evidence. Measurement reproducibility and clinical validity require evaluation within that context of use. A fixed cutoff is not a prerequisite when the candidate measure is analyzed as a continuous variable. A multilevel combination may serve as a mechanistic hypothesis or a candidate biomarker panel. However, before its definition has been locked and external replication and treatment-specific validation have been completed, it should not be characterized as a stable, independent, and generalizable clinical “phenotype” (27–29).

Operationally, PR is a modality-specific distal-to-proximal pressure ratio, commonly estimated with CTA-CFD in the cited sICAS cohorts. Angio-FF is an angiography-image-derived estimate used by its own software and acquisition workflow; it should not be renamed PR or assumed methodologically equivalent to pressure-wire fractional flow. CT-derived fractional flow reserve (CT-FFR) is a CT-based computational estimate, whereas digital-subtraction-angiography-derived pressure ratio (DPR) and magnetic-resonance-imaging-derived pressure ratio (MPR) are distinct modality-specific approaches. Each requires its own locked algorithm, measurement location, physiological conditions, and validation; values or thresholds are not interchangeable across modalities (Table 1).

**Table 1:** Operational definitions and threshold status of lesion-level hemodynamic measures.

Measurement	Imaging source	Formula or operational definition	Physiological construct	Direction of abnormality	Reported threshold and status
Pressure ratio (PR)	CTA-based CFD	PR = post-stenotic pressure/pre-stenotic pressure	Pressure preservation and residual antegrade flow across the stenosis	Lower PR indicates greater translesional pressure loss	The median value of 0.94 was used to define low PR; this was an internally derived, non-externally validated threshold (55).
Angiography-derived fractional flow (Angio-FF)	DSA	A computationally derived translesional pressure ratio from cerebral angiographic images, validated against pressure-wire FF	Angiography-estimated functional pressure loss across the lesion	Lower Angio-FF indicates more severe functional stenosis	Values of 0.70, 0.75, and 0.80 were used to identify corresponding pressure-wire FF thresholds; they are not recurrence-risk or treatment-selection thresholds (89).
CT-derived fractional flow reserve (CT-FFR)	Peak arterial-phase 4D-CTA plus CFD	Individualized CFD estimation of the pressure ratio distal to the stenosis; the distal sampling location and boundary conditions should be reported	Functional stenosis severity and distal pressure preservation	Lower CT-FFR indicates greater pressure loss	≤0.80; derived from CTP-hypoperfusion ROC analysis in a single-center cohort and internally validated only (57).
Wall shear stress (WSS)	CTA-CFD or 4D-flow MRI	Tangential mechanical stress acting on the vessel wall, calculated from the near-wall velocity gradient	Local vessel-wall mechanical environment, plaque biology, and vascular remodeling	No uniform abnormal direction; interpretation depends on the measurement site and summary statistic	No uniform clinical threshold; the use of maximum, mean, or high-WSS area should be specified (90).
Wall shear stress ratio (WSSR)	CTA-CFD	WSSR = WSS at the stenotic throat/pre-stenotic WSS	Local amplification of wall shear stress caused by stenosis	Higher WSSR indicates greater shear-stress amplification	High WSSR was defined as the highest quartile; this was an internally derived, non-externally validated threshold (55).
Relative mean transit time (rMTT)	CTP or perfusion MRI	Usually calculated as MTT in the affected target territory divided by MTT in the contralateral corresponding territory; perfusion software, deconvolution method, and ROI definition should be specified	Tissue-level delay in blood transit	Higher rMTT indicates delayed perfusion	No universal threshold; values depend on software, territory definition, and contralateral reference method.
Tmax delay volume	CTP or perfusion MRI	Volume of tissue within the target territory in which Tmax exceeds a prespecified threshold	Extent of tissue perfusion delay	Larger delay volume indicates a greater hypoperfusion burden	Tmax >6 s ≥ 15 mL has been used to define hypoperfusion in anterior-circulation ICAD; this is a study-specific operational definition, not a universally externally validated cut-off (58).
Quantitative magnetic resonance angiography (QMRA) flow	TOF-MRA localization plus 2D phase-contrast MRI	Volumetric flow (mL/min) is calculated from velocity measured in a plane perpendicular to the vessel axis	Large-vessel antegrade flow or regional blood supply	Lower flow indicates reduced antegrade blood supply	Thresholds depend on age-, vessel-, and territory-specific reference values; no universal clinical cut-off exists (64).
Collateral circulation score	DSA, single-phase CTA, or multiphase CTA	Ordinal grading based on the extent and delay of distal leptomeningeal vessel filling in the affected territory	Parallel blood-supply pathways and compensatory capacity	Reduced or delayed filling indicates poorer collateral circulation	No cross-modality universal threshold; the scoring system and definition of good versus poor collateral circulation should be specified (88).

CT, computed tomography; CTA, computed tomography angiography; 4D-CTA, four-dimensional computed tomography angiography; CFD, computational fluid dynamics; CTP, computed tomography perfusion; DSA, digital subtraction angiography; FF, fractional flow; ICAD, intracranial atherosclerotic disease; MRI, magnetic resonance imaging; MTT, mean transit time; ROC, receiver operating characteristic; ROI, region of interest; TOF-MRA, time-of-flight magnetic resonance angiography; Tmax, time to maximum of the tissue-residue function.

A candidate biomarker or biomarker combination that is ready to proceed to validation should specify at least six elements: the target population; measurement time point and physiologic conditions; algorithm, unit, and quality-control procedures for each component; rules for combining the components; acceptable extent of missing data; and intended context of use. “Stable” should denote acceptable reproducibility and transportability across centers under prespecified conditions. “Independent” should, at a minimum, indicate incremental value beyond existing clinical and imaging information within a prespecified model and should not be defined solely by a statistically significant *p* value in multivariable regression. “Generalizable” requires validation across centers, devices, algorithm versions, and patient populations.

### 3.2 Prognostic and predictive biomarkers and net clinical benefit

The FDA–NIH BEST framework defines a prognostic biomarker as a biomarker used to identify the likelihood of a disease-related event, recurrence, or progression, whereas a predictive biomarker identifies individuals who are more likely than comparable individuals without the biomarker to experience a favorable or unfavorable effect from a specific medical product or intervention (30). Accordingly, even when a low PR, a larger Tmax-delayed volume, or unfavorable venous outflow is associated with recurrent events, such a measure should initially be regarded as a candidate prognostic biomarker. It can be considered predictive only if it modifies the effect of treatment A relative to treatment B.

When the relative treatment effect is approximately constant across risk strata, a higher baseline risk generally results in a greater absolute risk reduction. Under these circumstances, a prognostic model may help determine whether treatment is likely to provide sufficient clinical benefit; stroke-risk scores used to guide anticoagulation thresholds in atrial fibrillation illustrate this principle (31, 32). In sICAS, however, balloon angioplasty, stenting, and medical therapy differ in peri-procedural risk, long-term benefit, and mechanisms of action. Therefore, a high risk of recurrence cannot automatically be interpreted as evidence of net benefit from an intervention. The clinically relevant quantity is the expected difference in outcome for an individual patient under treatment A versus treatment B after accounting for procedure-related stroke, hemorrhage, restenosis, and treatment burden (32, 33).

A treatment × biomarker interaction evaluates whether the treatment effect differs across biomarker levels, rather than whether treatment effects are statistically significant within the biomarker-positive and biomarker-negative groups separately. For a binary outcome, the absolute treatment effect can first be estimated within each stratum: RD_positive = risk_B − risk_A and RD_negative = risk_B − risk_A.

The additive interaction is then calculated as RD_positive − RD_negative. On the relative scale, risk ratios or odds ratios may be compared. For time-to-event outcomes, treatment, biomarker, and treatment × biomarker terms can be incorporated into a Cox proportional hazards model. Because conclusions regarding interaction may depend on the effect scale used, both absolute and relative treatment effects should be reported. The Predictive Approaches to Treatment effect Heterogeneity (PATH) Statement further recommends distinguishing risk modeling from treatment-effect modeling and cautions against interpreting one-variable-at-a-time *post hoc* subgroup analyses as evidence of individualized treatment effects (34, 35).

Accordingly, the evidence ladder required for a digital measure to progress from measurement validity to clinical use is summarized in Figure 1.

**Figure 1:**
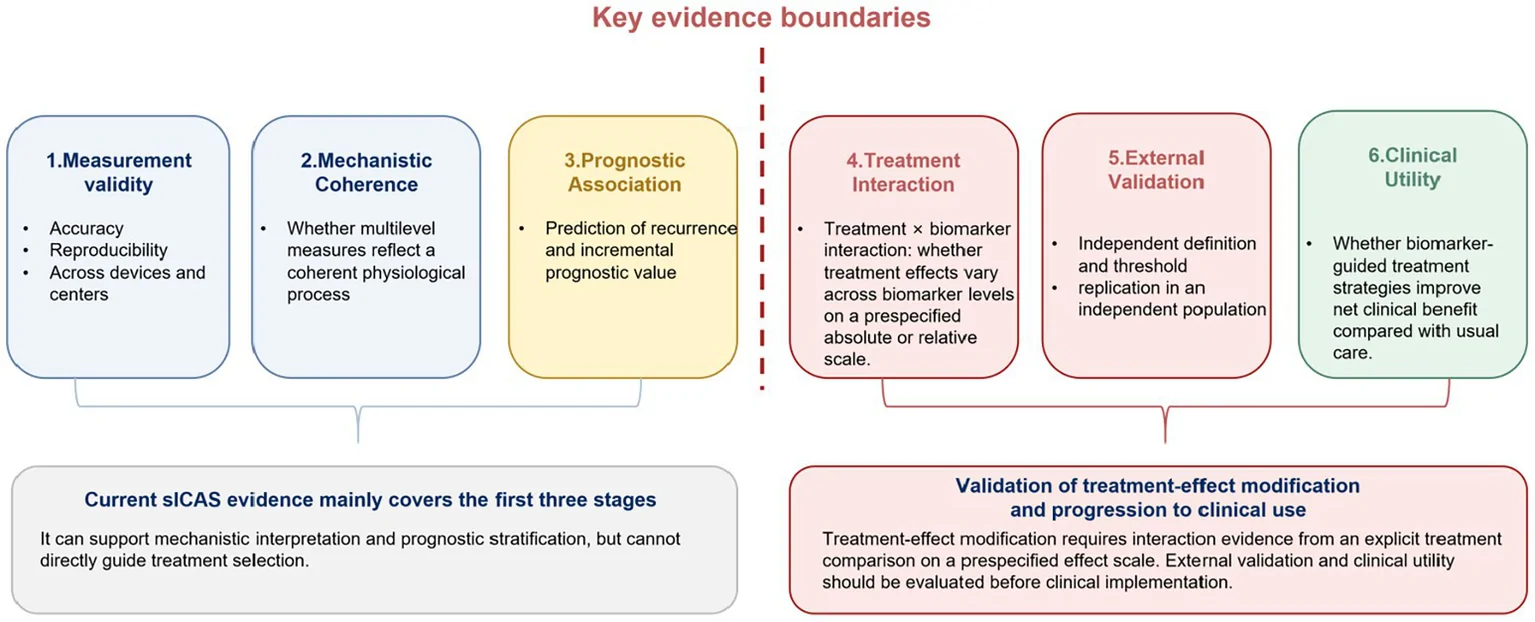
Evidence ladder from digital features to clinical utility. Current evidence in symptomatic intracranial atherosclerotic stenosis (sICAS) is concentrated at the stages of measurement and analytical validity, mechanistic coherence, and prognostic association, whereas evidence beyond the predictive-evidence boundary remains limited. Formal validation of a candidate predictive biomarker or panel requires an explicit treatment comparison and a prespecified treatment × biomarker interaction. Enrichment randomization can demonstrate treatment efficacy within a biomarker-defined population but cannot by itself establish effect modification across biomarker strata. External validation and demonstration of clinical utility are subsequently required.

## 4 Pathophysiologic framework of Hemodynamics in sICAS

### 4.1 Lesion function, distal perfusion, and compensatory mechanisms

Clinical practice already integrates symptom attribution, infarct distribution, culprit-lesion assessment, collateral status, perfusion, and vessel-wall imaging. Within this framework, digital hemodynamic measurements may provide additional value by improving measurement consistency, enhancing mechanistic interpretation, and potentially informing decision-related net clinical benefit. Based on the currently available direct evidence, the hemodynamics of sICAS can be conceptualized as one primary supply-chain pathway and two parallel injury pathways (Figure 2).

**Figure 2:**
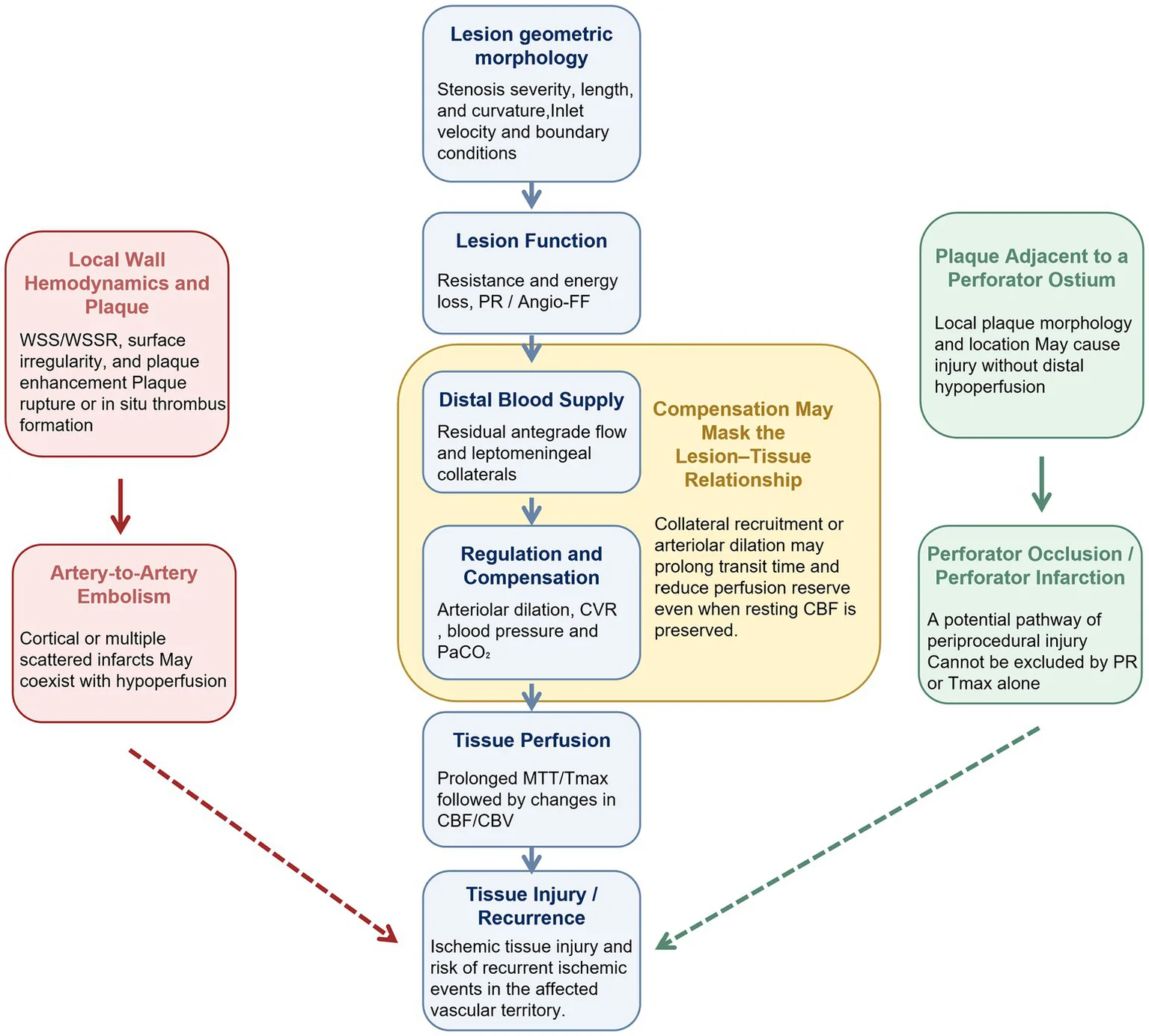
Conceptual model of cross-level pathophysiological relationships in sICAS. Solid arrows show direct same-cohort evidence or well-established physiological relations; dashed arrows show physiologically plausible or incompletely validated links. In the primary supply-chain pathway, lesion function, antegrade residual flow, collateral circulation, and autoregulation jointly influence tissue perfusion; local wall–embolism and perforator-ostial involvement represent parallel injury pathways. sICAS, symptomatic intracranial atherosclerotic stenosis; PR, pressure ratio; Angio-FF, angiography-derived fractional flow; WSS, wall shear stress; WSSR, wall shear stress ratio; CVR, cerebrovascular reserve; CBF, cerebral blood flow; CBV, cerebral blood volume; MTT, mean transit time; Tmax, time to maximum of the tissue-residue function; PaCO_2_, arterial partial pressure of carbon dioxide.

The primary supply-chain pathway can be understood as a sequential pathophysiological process. Lesion anatomy and geometry increase vascular resistance and energy loss, leading to impaired translesional pressure transmission and reduced antegrade residual flow. Distal perfusion, however, is not determined by antegrade flow alone. Collateral circulation and cerebrovascular autoregulation can compensate for reduced forward supply and help maintain tissue perfusion. When compensatory capacity becomes insufficient, prolongation of mean transit time (MTT) and Tmax may become evident, accompanied by changes in cerebral blood volume (CBV) and, with further hemodynamic decompensation, reductions in cerebral blood flow (CBF), tissue ischemia, characteristic infarct patterns, and an increased risk of recurrence.

This distinction is particularly important when interpreting PR and Angio-FF. These measures primarily reflect pressure loss across a stenotic lesion and do not directly quantify tissue perfusion or absolute cerebral blood flow. Although a lower PR generally indicates greater hemodynamic impairment at the lesion, patients with similar PR values may still have different degrees of distal hypoperfusion because collateral supply, distal vascular resistance, and cerebrovascular autoregulatory capacity vary among individuals. Therefore, lesion-level pressure impairment and tissue-level hypoperfusion should not be considered interchangeable, and a low PR cannot by itself be equated with hypoperfusion or borderzone infarction.

Lan et al. provided relatively direct same-cohort evidence supporting this concept in 83 patients with symptomatic M1 stenosis. Both PR derived from CTA-based computational fluid dynamics (CTA-CFD) and the leptomeningeal collateral score were independently associated with ipsilateral relative MTT (rMTT). In patients with moderate stenosis, the association between rMTT and PR was more prominent, whereas in those with severe stenosis, rMTT was more strongly associated with the extent of collateral circulation (36). These findings suggest that distal transit time is jointly influenced by antegrade residual flow and collateral compensation, and that their relative contributions may vary with stenosis severity. Thus, lesion-level pressure loss alone may not fully characterize distal perfusion status.

The coexistence of more extensive collateral circulation and prolonged rMTT in the same study also requires careful interpretation. Greater collateral recruitment should not be taken to mean that collateral flow itself causes hypoperfusion. A more plausible explanation is that insufficient antegrade flow promotes compensatory collateral recruitment. These collateral pathways may help preserve tissue supply, but the longer or more circuitous route of blood flow can still result in delayed arrival and prolonged transit time (36–38).

Cerebrovascular autoregulation further contributes to the potential mismatch between lesion-level hemodynamic measurements and tissue perfusion. When perfusion pressure declines, distal arteriolar dilation can reduce vascular resistance and temporarily preserve resting CBF, even when hemodynamic compromise is already present. This compensation may be accompanied by changes in CBV, prolongation of MTT/Tmax, and progressive depletion of cerebrovascular reserve. Consequently, preserved resting CBF does not exclude hemodynamic impairment. Conversely, an isolated prolongation of Tmax does not necessarily indicate irreversible low-flow ischemia, because Tmax may also be influenced by collateral pathways, systemic blood pressure, PaCO_2_, the timing of imaging relative to the acute event, and other physiological conditions (38).

These considerations help explain why PR, Tmax, QMRA flow, and cerebrovascular reserve measurements may be discordant across patients, imaging platforms, or time points. Such discordance may reflect different combinations of impaired antegrade flow, collateral compensation, and autoregulatory reserve. Accordingly, candidate hemodynamic biomarker combinations should prespecify concordant and discordant patterns across measurements, rather than simply summing the number or severity of abnormal findings.

### 4.2 Infarct patterns and ischemic mechanisms

The major ischemic mechanisms of sICAS include artery-to-artery embolism, perforator ostial involvement, hypoperfusion, and mixed mechanisms. In the WASID imaging analysis, artery-to-artery embolic patterns were the most common, whereas isolated hypoperfusion was relatively uncommon (39). In another study of 153 patients, hypoperfusion and the mixed mechanism of artery-to-artery embolism combined with hypoperfusion were also frequently observed. Among patients receiving medical therapy, the mixed mechanism was associated with a higher 1-year risk of recurrent ischemic events in the same vascular territory (24.4% vs. 7.8%; HR, 3.40; 95% CI, 1.25–9.20) (40). These findings suggest that hypoperfusion does not represent a universal downstream pathway for all sICAS-related ischemic events and that artery-to-artery embolism and perforator involvement should be considered parallel pathogenic mechanisms.

Recent studies have further clarified the relationship between perfusion abnormalities and infarct patterns. In a study of 42 patients with acute sICAS, internal borderzone infarction was associated with larger Tmax >4 s and Tmax >6 s delayed volumes and greater perfusion mismatch than branch-occlusive mechanisms. However, the artery-to-artery embolic subtype could also exhibit a relatively large Tmax >6 s volume, indicating that marked perfusion delay is not specific to a purely hypoperfusion-mediated mechanism (41). In another study of 84 patients with symptomatic M1 stenosis and borderzone infarction, a low PR was independently associated with isolated internal borderzone infarction, whereas cortical borderzone infarction was more frequently accompanied by small cortical infarcts, suggesting a potential embolic component (42). Similarly, a 2025 study of 144 patients showed that borderzone or territorial infarction was associated with more pronounced perfusion impairment than perforator infarction, and a Tmax >4 s mismatch volume >15 mL was associated with poorer early neurological outcomes (43). Collectively, these findings support the view that internal borderzone infarction is more closely related to the distal consequences of impaired cerebral perfusion, while also underscoring that neither borderzone infarction nor Tmax abnormality should be regarded as a specific marker of pure hypoperfusion.

### 4.3 Local wall hemodynamics and embolic and perforator mechanisms

Plaque enhancement, positive remodeling, T1 hyperintensity, and surface irregularity on high-resolution vessel-wall MRI have been associated with culprit lesions or recurrent ischemic events (44–48). Local WSS, wall shear stress ratio (WSSR), and plaque characteristics primarily reflect the local mechanical environment of the vessel wall and plaque stability. These features are therefore more relevant to local plaque-related thrombosis and embolic mechanisms and should not be regarded as obligatory intermediate variables linking translesional pressure loss to distal tissue hypoperfusion. In a study of 110 patients with middle cerebral artery atherosclerosis, distinct combinations of WSS and plaque characteristics were observed across asymptomatic lesions, artery-to-artery embolic mechanisms, and local branch-occlusive mechanisms (49), further supporting the role of local wall hemodynamics and plaque characteristics in a local injury pathway that runs in parallel with the primary supply pathway.

Perforator-ostial involvement constitutes another relatively independent local injury pathway. A 2026 study of 39 patients combining vessel-wall imaging (VWI) and optical coherence tomography (OCT) found that plaques associated with perforator infarction more frequently exhibited intraplaque hemorrhage, ulceration, a thin fibrous cap, and surface thrombus (50). These findings suggest that local plaque morphology and vulnerability may be related to perforator-ostial involvement. However, the small sample size, cross-sectional design, and invasively selected study population limit these findings primarily to mechanistic differentiation and do not support conclusions regarding prognosis or treatment-effect modification. Perforator-ostial involvement may also bypass the distal tissue-perfusion pathway and directly produce perforator infarction and may contribute to peri-procedural ischemic injury during stenting or balloon angioplasty.

Although hypoperfusion, artery-to-artery embolism, and perforator involvement can be analyzed as distinct mechanisms, these pathways are not necessarily independent. From a physiological perspective, hypoperfusion resulting from impaired lesion function may reduce the clearance of distal microemboli, thereby increasing the likelihood of embolic retention and recurrent ischemia and creating a mutually reinforcing relationship between low flow and impaired embolic washout (51, 52). However, among the direct sICAS studies identified in this review, no longitudinal mediation analysis has validated this complete pathophysiological process. This relationship should therefore remain a physiologically plausible mechanistic hypothesis rather than be interpreted as an established longitudinal causal pathway. The direct evidence supporting these proposed pathophysiological relationships, the interpretations supported by the available data, and the corresponding limits of inference are summarized in Table 2.

**Table 2:** Direct evidence supporting key pathophysiological relationships in sicas and their interpretive boundaries.

Proposed pathophysiological relationship	Key direct evidence	Interpretation supported by current evidence	Limit of inference
Lesion functional impairment and collateral status in relation to distal transit time	Lan et al., 2020 (*n* = 83): both PR and leptomeningeal collateral score were independently associated with ipsilateral rMTT, and the relative strength of these associations differed according to stenosis severity (36).	Distal transit time is jointly influenced by antegrade residual flow and collateral compensation; their relative contributions may vary with stenosis severity.	PR should not be assumed to correspond one-to-one with tissue perfusion, and greater collateral extent should not be interpreted as a cause of hypoperfusion.
Perfusion impairment and infarct pattern	Kim et al., 2021; Li et al., 2024; Xu et al., 2025: internal borderzone infarction was more frequently associated with impaired perfusion or lower PR, whereas embolic patterns could also show substantial Tmax abnormalities (41–43).	Internal borderzone infarction is more closely associated with impaired distal perfusion, while infarct pattern provides probabilistic information about the underlying ischemic mechanism.	Neither a Tmax abnormality nor the presence of a borderzone infarct is specific for a pure hypoperfusion mechanism.
Local wall hemodynamics and plaque characteristics in relation to embolic mechanisms	Woo et al., 2023 (*n* = 110): distinct combinations of WSS and plaque characteristics were observed across asymptomatic lesions, artery-to-artery embolism, and local branch-occlusive mechanisms (49).	Local wall hemodynamics and plaque characteristics may contribute to plaque instability, thrombosis, and artery-to-artery embolic mechanisms that operate in parallel with the distal perfusion pathway.	WSS/WSSR should not be assumed to constitute a necessary causal step between translesional pressure loss and distal perfusion abnormalities.
Plaque characteristics and perforator involvement	Xue et al., 2026 (*n* = 39): plaques associated with perforator infarction more frequently showed intraplaque hemorrhage, ulceration, thin fibrous caps, and surface thrombus (50).	Plaque morphology and vulnerability may help distinguish perforator-related mechanisms from other ischemic mechanisms.	The small, cross-sectional, invasively selected cohort does not establish prognostic value, treatment-effect modification, or direct treatment-selection utility.
Distal perfusion impairment and recurrent ischemic events	Single-center cohorts, a borderzone infarction meta-analysis, and the multicenter BIORISK ICAS study reported associations of Tmax abnormalities or borderzone infarction with recurrent ischemic events (58–61).	Distal perfusion abnormalities have prognostic value and may be useful for risk enrichment and for evaluating multivariable hemodynamic biomarker combinations.	Current evidence does not establish hypoperfusion as a predictive biomarker of benefit from balloon angioplasty or stenting.

sICAS, symptomatic intracranial atherosclerotic stenosis; ICAS, intracranial atherosclerotic stenosis; PR, pressure ratio; rMTT, relative mean transit time; WSS, wall shear stress; WSSR, wall shear stress ratio; Tmax, time to maximum of the tissue-residue function; BIORISK ICAS, Biomarkers and Recurrence Risk in Symptomatic Intracranial Atherosclerosis.

## 5 Clinical evidence for hemodynamic biomarkers in sICAS

### 5.1 Criteria for classifying prognostic and predictive evidence

Clinical studies are classified using the prognostic–predictive distinction defined in Section 3.2. Accordingly, findings such as “prediction of recurrence” in a cohort study, an independent association in a regression model, or improvement in the C statistic or area under the curve (AUC) provide evidence of prognostic value only. Similarly, in a single-arm interventional study, an association between a pretreatment biomarker and subsequent recurrence remains prognostic within that treatment setting. Evidence for predictive value requires comparison between alternative treatments, preferably within a randomized design, together with a prespecified test of the treatment × biomarker interaction and independent replication of the finding.

Prognostic enrichment preferentially enrolls patients at higher expected event risk to improve trial efficiency. A randomized trial restricted to a biomarker-defined population can establish treatment efficacy within that population, but cannot by itself demonstrate that treatment effects differ across biomarker strata (30).

### 5.2 Prognostic value of lesion function, distal perfusion, and compensatory measures

At the lesion level, PR and CT-derived fractional flow reserve (CT-FFR) primarily reflect translesional pressure transmission, whereas WSSR characterizes the local mechanical environment (53, 54). In a cohort of 245 patients with sICAS, the 1-year rate of recurrent ischemic events in the same vascular territory was 17.5% among patients with concomitantly low PR and high WSSR, compared with 3.0% among those with normal hemodynamic status; the adjusted hazard ratio (HR) was 7.52 (55). The same source cohort was subsequently used to develop the D_2_H_2_A nomogram, which assigned 2 points for diabetes, 5 points for dyslipidemia, 7 or 10 points for intermediate or abnormal hemodynamic status, respectively, 1 point for hypertension, and 3 points for age ≥50 years. Hemodynamic status was jointly defined by PR and WSSR: normal, intermediate, and abnormal states corresponded to both measures being normal, one measure being abnormal, and both measures being abnormal, respectively. The study-specific thresholds were PR at or below the median and WSSR at or above the fourth quartile. The model was developed in the same cohort of 245 patients, of whom 20 (8.2%) experienced 1-year same-territory recurrence, and therefore represents model development within the source dataset.

The D_2_H_2_A model had a C statistic of 0.73 (95% CI, 0.60–0.85) (56). This can be interpreted approximately as follows: if one patient who experienced the outcome and one who did not are randomly selected, the model has about a 73% probability of assigning a higher predicted risk to the patient who experienced the event. This does not mean that 73% of individual patients were classified correctly, nor does it imply a 73% benefit from stenting or balloon angioplasty. The C statistic evaluates discrimination only. Clinical application of a prognostic model additionally requires assessment of calibration, external validation, decision-curve analysis, and implementation (27, 28, 33). Using such a prognostic model to inform treatment selection requires integrating calibrated risk estimates with reliable evidence on comparative treatment effects and harms, followed by evaluation of clinical utility in the intended population. Any claim that the model identifies treatment-effect modification additionally requires supporting evidence from prespecified interaction analyses, preferably within randomized treatment comparisons.

In a 2026 retrospective study of 124 patients with symptomatic middle cerebral artery (MCA) stenosis, CT-FFR ≤ 0.80 was associated with 1-year recurrence (adjusted HR, 2.97), with a cross-validated AUC of 0.72 (57). Collectively, these studies support the prognostic value of lesion-related hemodynamic measures. Their common limitations include single-center or source-dataset designs, dependence on specific computational models, and thresholds derived from the study data. A systematic review of 19 CFD studies similarly concluded that these approaches are promising, while emphasizing substantial heterogeneity in boundary conditions, rheological assumptions, and validation methods and the need for standardization and multicenter validation (26).

Evidence based on distal tissue perfusion is more directly related to the pathway leading to clinical ischemic events. In a study of 50 patients with symptomatic anterior-circulation intracranial atherosclerotic disease (ICAD), a Tmax >6 s volume ≥15 mL was associated with 90-day recurrence (HR, 6.80) (58). In a cohort of 204 patients receiving optimized medical treatment, each 10-mL increase in Tmax >4 s volume was associated with a higher 1-year risk of same-territory stroke or vascular death (adjusted HR, 1.028), although the 83-mL threshold was derived from the same cohort (59). A meta-analysis of eight studies involving 1,219 patients found that borderzone infarction was associated with a higher risk of recurrent cerebrovascular events or neurological deterioration (risk ratio, 2.10) (60). In 2026, the BIORISK ICAS study included 1,891 patients in its main analysis across 35 centers. Borderzone infarction was associated with 90-day same-territory recurrence (adjusted HR, 1.40), and among the 509 patients in the perfusion subgroup, a Tmax >6 s mismatch volume ≥10 mL was also associated with recurrence (adjusted HR, 1.83) (61). This study currently provides one of the most representative multicenter data sets supporting the prognostic value of distal hypoperfusion, although it did not compare outcomes between different treatment strategies.

Negative and inconsistent findings also indicate that the prognostic performance of individual hemodynamic measurements is not uniformly reproducible. In the prospective multicenter MyRIAD study, prespecified measures of QMRA, perfusion, cerebrovascular reactivity, and microembolic signals were not significantly associated with clinical recurrence (62). Imaging patterns of recurrent infarction in the same study also demonstrated substantial heterogeneity in the underlying ischemic mechanisms (63). In a subsequent analysis of 73 patients with anterior-circulation disease, a redefined regional QMRA low-flow criterion identified recurrence rates of 21% versus 4%, with an age-adjusted odds ratio (OR) of 7.2. However, the confidence interval was wide (1.2–43.2), and the threshold had been optimized *post hoc* (64). Evidence for venous outflow is currently limited to a retrospective cohort of 99 patients with severe intracranial stenosis or occlusion (65). Venous outflow should therefore, at most, be considered an exploratory integrated terminal readout or a component of sensitivity analyses, rather than a necessary element of the core biomarker combination.

### 5.3 Candidate biomarkers for treatment-effect prediction: current evidence and limitations

A *post hoc* analysis of SAMMPRIS showed that, among patients receiving aggressive medical management, 14 of 53 patients with anterior-circulation borderzone infarction experienced recurrent ischemic events, with the highest recurrence risk (37%) observed in those with both borderzone infarction and impaired collateral circulation (66). However, within the borderzone-infarction subgroup, 10 of 55 patients in the stenting group and 14 of 53 in the medical-management group experienced recurrent events, corresponding to an HR of 0.65 (95% CI, 0.29–1.47; *p* = 0.30) (66). Given the *post hoc* design and limited sample size, this finding remains hypothesis-generating.

For predictive-biomarker assessment, the relevant BASIS finding is that the prespecified treatment × hypoperfusion interaction was not significant (*p* = 0.99) (16). Sparse data in the non-hypoperfusion stratum make this interaction estimate imprecise, but the trial did not validate hypoperfusion as a treatment-effect modifier. Hypoperfusion may remain useful for prognostic enrichment, not for selecting balloon angioplasty.

Mechanism-based patient exclusion has not resolved the treatment-selection problem. In SAMMPRIS, most peri-procedural ischemic events were perforator-territory infarctions and frequently occurred after technically successful basilar-artery angioplasty, supporting a procedure-related injury mechanism involving perforator ostial compromise or a “snow-plow” effect (67). In a 2025 single-center randomized trial that excluded patients with adjacent branch atheromatous disease, the primary outcome occurred in 16% of the stenting group and 24% of the medical-treatment group (HR, 0.66; 95% CI, 0.32–1.36; *p* = 0.26) (68). These findings indicate that excluding one recognized mechanism of procedural harm was still insufficient to demonstrate a clear benefit of stenting in the remaining population. Similarly, a pooled individual-patient-data analysis of 809 participants from SAMMPRIS and CASSISS found that the overall 30-day rate of stroke or death remained higher with stenting than with medical treatment (10.5% vs. 4.2%; HR, 2.62) (69).

### 5.4 Major clinical evidence, 2019–2026

Table 3 summarizes the evidentiary classification and population scope of lesion-function, perfusion, compensatory, and parallel-pathway measures. Among the completed and reported direct sICAS studies identified in this review, no candidate biomarker combination has yet crossed the threshold from prognostic evidence to validated predictive evidence for treatment selection.

**Table 3:** Major direct evidence from 2019 to 2026 and biomarker classification.

Study/design	Measure or candidate biomarker	Main result	Evidence classification	Predictive-evidence gap	Population scope
Leng 2019; cohort *n* = 245 (55)	CTA-CFD: PR, WSSR	Both abnormal: 17.5% vs. 3.0%; aHR 7.52.	Prognostic; lesion-level mechanism	Within-cohort thresholds; no treatment comparison.	Direct sICAS cohort
Lan 2020; cross-level cohort *n* = 83 (36)	PR + leptomeningeal collaterals + rMTT	PR and collaterals were both independently associated with rMTT; relationships changed with stenosis severity.	Lesion-compensation-tissue cross-level mechanism	Cross-sectional association; no longitudinal mediation or treatment comparison.	Direct symptomatic M1 stenosis
Kim 2021; mechanistic cohort *n* = 42 (41)	Infarct mechanism + Tmax/mismatch	Internal borderzone infarction had the greatest perfusion delay; embolic type could also show Tmax abnormalities.	Mechanistic association; suggests mixed pathways	Perfusion delay is not specific to pure hypoperfusion.	Direct acute sICAS cohort
Wabnitz 2019; SAMMPRIS *post hoc* (62)	Borderzone infarction + impaired collaterals	Highest recurrence in medical group: 37%; stenting vs. medical therapy within borderzone subgroup HR 0.65, *p* = 0.30.	Strong prognostic signal; predictive evidence unproven	Post hoc, small sample; no reliable replicated interaction.	Direct SAMMPRIS subset with symptomatic intracranial ICA or MCA infarction
de Havenon 2020; retrospective *n* = 50 (58)	Tmax >6 s volume ≥15 mL	90-day recurrence HR 6.80.	Prognostic	Small sample; threshold requires external validation.	Retrospective cohort of hospitalized patients with symptomatic intracranial ICA or MCA atherosclerotic stenosis (50–99%).
MyRIAD 2021; prospective multicenter *n* = 105 (62)	QMRA, perfusion-weighted imaging, vasomotor reactivity, microembolic signals	Prespecified measures were not significantly associated with clinical recurrence.	Negative prespecified prognostic evidence	Suggests that simple parallel listing does not form a stable combination.	Direct multicenter sICAS cohort
Tian 2023; model development *n* = 245 (56)	D_2_H_2_A + CFD status	Internal C statistic 0.73 (95% CI, 0.60–0.85).	Prognostic model development	Source dataset; lacks external validation and treatment interaction.	Direct sICAS cohort; model developed in the 245-patient source dataset
Das 2023; systematic review *n* = 1,219 (60)	Borderzone infarction	Recurrence/deterioration RR 2.10.	Prognostic; cross-study consistency	Infarct pattern is not evidence of treatment-effect modification.	Systematic review of sICAS cohorts; population definitions varied across included studies
Gao 2023; retrospective *n* = 99 (65)	Unfavorable venous outflow	Independently associated with 1-year recurrence.	Exploratory prognostic biomarker	Mixed stenosis and occlusion; no stable threshold.	Related mixed severe ICAS/occlusion cohort
Yang 2024; validation *n* = 121 (89)	Angiographic functional parameters	Correlation with pressure wire *r* = 0.92; AUC 0.962–0.986.	Measurement/analytical validity	Cannot infer validity for outcomes or treatment selection.	ICAS measurement-validation cohort; symptom status not specified
Li 2024; CFD cohort *n* = 84 (42)	PR + internal/cortical borderzone pattern	Low PR was independently associated with isolated internal borderzone infarction; cortical type more often accompanied small cortical infarcts.	Lesion function-infarct pattern mechanism	Borderzone classification is not a completely specific causal label.	Direct symptomatic MCA-M1 sICAS cohort
Huang 2024; PTAS cohort *n* = 137 (91)	qDSA rTTP	Pretreatment rTTP was associated with post-treatment recurrence; incremental AUC 0.817.	Prognostic within an interventional setting	All patients underwent PTAS; relative treatment effect cannot be estimated.	Related post-PTAS intracranial vertebrobasilar atherosclerotic stenosis cohort
BASIS 2024; RCT *n* = 501 (16)	Hypoperfusion subgroup	Hypoperfusion: 3.9% vs. 15.1%, HR 0.25; P for interaction = 0.99.	Overall balloon efficacy positive; predictive evidence unproven	Within-subgroup effect is not interaction; very few events in the non-hypoperfusion stratum, making the interaction estimate imprecise.	Direct RCT-defined 70–99% sICAS cohort
Yu 2026; prospective *n* = 204 (59)	Tmax >4 s volume	Per 10 mL: aHR 1.028; internal threshold 83 mL.	Prognostic	Single-cohort threshold; no treatment comparison.	Prospective cohort of patients with ischemic stroke within 30 days attributable to 50–99% intracranial atherosclerotic stenosis, all receiving optimal medical treatment.
Xu 2025; cohort *n* = 144 (43)	Perfusion + infarct pattern	Borderzone/territorial infarction had greater perfusion impairment; Tmax >4 s mismatch >15 mL was associated with poorer early outcome.	Tissue-level mechanism/early prognosis	Not recurrence prediction or treatment interaction.	Direct anterior-circulation sICAS cohort (50–99% stenosis)
Amin-Hanjani 2025; MyRIAD post hoc *n* = 73 (64)	Regional QMRA low flow	21% vs. 4%; age-adjusted OR 7.2.	Prognostic hypothesis generation	Post hoc threshold; wide CI.	Direct MyRIAD anterior-circulation sICAS subgroup
Ip 2025; single-center RCT *n* = 150 (68)	Exclusion of adjacent branch disease	Stenting 16% vs. medical therapy 24%; HR 0.66, *p* = 0.26.	Negative mechanism-selection trial	Excluding perforator risk is insufficient to establish benefit from stenting.	Direct high-grade sICAS RCT cohort; adjacent branch atheromatous disease excluded
BIORISK 2026; 35 centers *n* = 1,891 (61)	Borderzone infarction; Tmax >6 s mismatch	aHR 1.40; perfusion subgroup *n* = 509, aHR 1.83.	Relatively strong multicenter prognostic evidence	No randomized treatment comparison; threshold still requires locking.	Direct multicenter international sICAS cohort (50–99% stenosis)
Yin 2026; retrospective *n* = 124 (57)	CT-FFR ≤ 0.80	1-year recurrence aHR 2.97; cross-validated AUC 0.72.	Prognostic; functional measure	Single center; threshold and platform dependent.	Single-center retrospective cohort of patients with symptomatic unilateral MCA-M1 stenosis (30–99%), including patients with stenosis <50%.

sICAS, symptomatic intracranial atherosclerotic stenosis; ICAD, intracranial atherosclerotic disease; MCA, middle cerebral artery (M1, first segment); PR, pressure ratio; Angio-FF, angiography-derived fractional flow; CT-FFR, computed tomography-derived fractional flow reserve; CTA/CFD, computed tomography angiography/computational fluid dynamics; WSSR, wall shear stress ratio; rMTT/rTTP, relative mean transit time/time to peak; QMRA, quantitative magnetic resonance angiography; qDSA, quantitative digital subtraction angiography; PTAS, percutaneous transluminal angioplasty and stenting; HR/aHR, hazard ratio/adjusted hazard ratio; CI, confidence interval; OR/RR, odds ratio/risk ratio; AUC, area under the receiver operating characteristic curve; RCT, randomized controlled trial; Tmax, time to maximum of the tissue-residue function; D_2_H_2_A, diabetes, dyslipidemia, hemodynamic status, hypertension, and age nomogram; SAMMPRIS, Stenting and Aggressive Medical Management for Preventing Recurrent Stroke in Intracranial Stenosis; BASIS, Balloon Angioplasty for Symptomatic Intracranial Artery Stenosis; BIORISK ICAS, Biomarkers and Recurrence Risk in Symptomatic Intracranial Atherosclerosis. The Population scope column identifies the applicability boundary for each study. Related/mixed cohorts are included for mechanistic or prognostic context and are not direct treatment-selection evidence for strictly defined sICAS.

## 6 Candidate biomarkers for treatment-effect prediction and their validation priorities

For endovascular therapy, candidate treatment-effect prediction should jointly assess two relatively independent dimensions. The potential-benefit axis comprises impaired lesion function, distal hypoperfusion or regional low flow, and failed compensation, indicating a potentially reversible hemodynamic burden. The potential-harm axis comprises perforator-ostial involvement, qualifying-infarct mechanism, lesion location or geometry, and risks of peri-procedural hemorrhage or restenosis. The aim is to estimate net clinical benefit for a specified treatment comparison, not positivity on either axis alone.

Cross-level combinations do not require all measurements to be abnormal. Their prespecified interpretation should address concordant and discordant patterns: preserved perfusion despite lesion-level impairment may reflect effective compensation, whereas impaired perfusion without a corresponding lesion-level abnormality warrants reassessment of alternative inflow, collateral, distal, or nonculprit mechanisms. Candidate benefit biomarkers, harm modifiers, and their validation priorities are summarized in Table 4. Because the balance between potential benefit and procedural harm differs by intervention, a biomarker combination proposed for stenting cannot be assumed to predict benefit from balloon angioplasty or medical therapy. Candidate indicators should therefore be specified for a particular treatment comparison.

**Table 4:** Treatment-specific candidate benefit biomarkers, harm modifiers, and falsifiable research hypotheses.

Candidate biomarker/modifier	Candidate minimum data combination	Candidate research comparison and mechanistic rationale	Testable hypothesis and falsification criterion	Current evidence and requirements for further validation
1A Angio-FF lesion-function enrichment biomarker	DSA-derived Angio-FF; locked software, acquisition conditions, core laboratory, and ≤0.70 threshold; record distal perfusion, infarct pattern, and collaterals for mechanistic stratification.	Candidate comparison: stenting + aggressive medical management vs. aggressive medical management. Impaired translesional function may identify patients more likely to benefit from luminal reconstruction.	Hypothesis to be tested: Angio-FF may modify the treatment effect of stenting. Predictive validity requires a prespecified treatment × biomarker interaction across Angio-FF strata and independent replication	Angio-FF has entered prospective randomized evaluation in the ICAS-MT registered design, but results are not yet available and the current design does not randomize patients across the full Angio-FF range (70). Further validation should report the proportion of evaluable Angio-FF measurements and reasons for non-evaluable cases, assess cross-center reproducibility, and determine its incremental value beyond established clinical and anatomical information. The locked algorithm and prespecified threshold should also be evaluated in independent populations. When Angio-FF is modeled together with other lesion-function measures, inter-measure correlations and information redundancy should be assessed.
1B Distal hemodynamic decompensation combination (benefit axis)	Lesion-function measure (PR/FF/CT-FFR) + distal measure (Tmax hypoperfusion volume or regional QMRA low flow); use collateral findings to support assessment of compensatory status and infarct patterns to characterize likely ischemic mechanisms, including possible perforator involvement.	Candidate comparison: balloon angioplasty or stenting plus aggressive medical management versus aggressive medical management alone, evaluated separately. Lesion-level functional impairment may further compromise distal tissue perfusion when collateral and autoregulatory compensation is inadequate, providing a mechanistic rationale for testing luminal reconstruction.	Hypothesis to be tested: greater distal hemodynamic decompensation may be associated with a differential effect of luminal reconstruction. The hypothesis would receive little support if the prespecified treatment × continuous-biomarker interaction is negligible or peri-procedural harm offsets later benefit.	Prognostic and mechanistic evidence for distal hypoperfusion is comparatively strong, whereas direct evidence for treatment-effect prediction remains limited. BASIS did not demonstrate significant effect modification by hypoperfusion (interaction *p* = 0.99), and the small number of events in the non-hypoperfusion stratum limited the precision of the interaction estimate (16). Further studies should prespecify rules for selecting and combining lesion-function and distal hemodynamic measures, assess information redundancy and model stability, and report missingness for each component and the proportion of patients with complete multimodal data. Incremental value should be evaluated against established clinical and conventional imaging models, followed by validation in independent multicenter cohorts.
2 Blood-pressure-dependent low-flow/reserve-exhaustion combination	Regional low flow or Tmax abnormality + impaired cerebrovascular reserve; record blood pressure, PaCO_2_, and repeated measurements at the time of assessment; if necessary, observe state responses to changes in posture/blood pressure.	Candidate research comparison: a prespecified gradual blood-pressure-lowering strategy versus guideline-based management in a randomized mechanistic safety study. When perfusion pressure approaches the lower limit of cerebral autoregulation, further reductions in perfusion pressure may affect cerebral blood flow.	Hypothesis to be tested: low-flow/reserve-exhaustion status may modify the effects of blood-pressure reduction. The hypothesis would receive little support without a prespecified treatment × biomarker interaction or reproducible differential hemodynamic response.	The hypothesis is physiologically plausible, but direct predictive evidence in sICAS remains weak. VERiTAS provides observational evidence from an adjacent vertebrobasilar disease population (71–73), and current evidence is insufficient to support modification of guideline-recommended blood-pressure targets. Further studies should assess information overlap among Tmax, QMRA flow, and CVR; prespecify the handling of patients unable to complete CVR or repeated physiological testing; and determine whether blood pressure, PaCO_2_, posture, vasoactive medications, and measurement timing can be standardized across centers. The incremental predictive value of low-flow/CVR measures and the clinical utility of biomarker-guided blood-pressure strategies require prospective evaluation.
3 Perforator-risk combination (harm axis)	Qualifying perforator-territory infarction + plaque adjacent to a perforator ostium or branch atheromatous disease + absence of dominant distal hypoperfusion.	Candidate comparison: stenting or balloon angioplasty versus medical therapy. Device expansion may displace plaque and compromise perforator ostia, consistent with a potential “snow-plow” mechanism.	Hypothesis to be tested: a prespecified perforator-risk profile may modify the peri-procedural effect of intervention, particularly perforator stroke risk. The hypothesis would receive little support if no treatment × biomarker interaction is observed.	SAMMPRIS provides evidence supporting a perforator-related mechanism of peri-procedural injury, although predictive value for treatment selection has not been established. A randomized trial excluding adjacent branch disease also failed to demonstrate a clear benefit of stenting in the remaining population (67, 68). Further validation should prespecify the definition of the perforator-risk combination, assess information overlap among its components, report the availability and non-evaluable cases for VWI and angiographic branch assessment, and examine inter-reader agreement and cross-center reproducibility. Its incremental value beyond lesion location, stenosis severity, and conventional infarct-pattern assessment should also be determined and externally validated.
4 Active plaque-embolism combination (exploratory)	VWI enhancement/surface irregularity + cortical or multiple scattered infarcts/microembolic signals; WSS/WSSR as support for the local environment.	Candidate comparison: intensified short-term antithrombotic or plaque-stabilizing therapy versus standard therapy. Markers of plaque activity and embolization may identify a pathological pathway more responsive to intensified medical treatment.	Hypothesis to be tested: an active plaque–embolism profile may modify the effect of intensified antithrombotic or plaque-stabilizing therapy. The hypothesis would receive little support without a prespecified treatment × biomarker interaction or if bleeding harm offsets ischemic benefit.	Prognostic and mechanistic evidence is available (40, 45, 46), but direct randomized treatment comparisons and formal biomarker interaction analyses remain limited. Further studies should prespecify the selection and combination of VWI features, infarct patterns, microembolic signals, and WSS/WSSR; assess information overlap; and report the availability and missingness of each modality. The feasibility burden of the complete multimodal protocol and its incremental predictive value beyond standard clinical and imaging information should also be assessed. Prospective treatment comparisons should jointly evaluate ischemic benefit and treatment-related harm.

sICAS, symptomatic intracranial atherosclerotic stenosis; PR, pressure ratio; FF, fractional flow; Angio-FF, angiography-derived fractional flow; CT-FFR, computed tomography-derived fractional flow reserve; DSA, digital subtraction angiography; QMRA, quantitative magnetic resonance angiography; CVR, cerebrovascular reserve; VWI, vessel-wall imaging; WSS, wall shear stress; WSSR, wall shear stress ratio; PaCO_2_, arterial partial pressure of carbon dioxide; ICAS-MT, Intracranial Angiography-Derived Fractional-Flow-Guided Percutaneous Transluminal Angioplasty and Stenting Versus Medical Therapy; SAMMPRIS, Stenting and Aggressive Medical Management for Preventing Recurrent Stroke in Intracranial Stenosis; BASIS, Balloon Angioplasty for Symptomatic Intracranial Artery Stenosis.

The same biomarker may have different implications across treatment strategies. Distal hemodynamic decompensation may increase the potential benefit of restoring antegrade flow but does not necessarily offset the perforator-related procedural risk of stenting. Conversely, a perforator-risk profile may reduce the net clinical benefit of stenting without predicting the effect of balloon angioplasty or medical therapy. Candidate indicators should therefore be specified and validated for a particular treatment comparison.

## 7 Study designs for biomarkers of treatment-effect prediction

### 7.1 Angio-FF-enriched randomized design of ICAS-MT

The IntraCranial Angiography-derived Fraction Flow-guided Percutaneous Transluminal Angioplasty and Stenting Versus Medical Therapy (ICAS-MT) study is a registered multicenter, prospective, open-label study with blinded endpoint assessment and a planned enrollment of 536 patients (70). Of these, 336 patients with Angio-FF ≤ 0.70, defined as having hemodynamic impairment, are planned to undergo randomized comparison of stenting plus aggressive medical management versus aggressive medical management alone, whereas 200 patients with Angio-FF > 0.70 are planned to be enrolled in a registry cohort. The primary endpoint is stroke or death within 30 days after enrollment, or stroke in the territory of the qualifying artery between 30 days and 1 year. Major eligibility criteria include age 30–80 years, transient ischemic attack (TIA) or ischemic stroke within the preceding 3 months attributable to 70–99% stenosis of the intracranial internal carotid artery, M1 segment of the middle cerebral artery, V4 segment of the vertebral artery, or basilar artery, and a modified Rankin Scale (mRS) score ≤2. For patients presenting with stroke, stenting is scheduled more than 2 weeks after the most recent qualifying event (70).

The importance of this design lies in placing Angio-FF–based functional screening before randomization to stenting in patients with sICAS. It is therefore intended to address a specific treatment question: among patients who meet all trial eligibility criteria and have Angio-FF ≤ 0.70, does stenting plus aggressive medical management provide greater benefit than aggressive medical management alone? Compared with enrollment based solely on 70–99% anatomic stenosis, this strategy more closely reflects a mechanistic-enrichment approach. If the primary endpoint favors stenting and the accompanying peri-procedural risk, long-term same-territory stroke risk, absolute risk reduction, and robustness of the findings are all acceptable, Angio-FF ≤ 0.70 could become a candidate enrollment threshold supported by randomized evidence within this specific patient population and technical setting, and could inform future clinical screening strategies.

### 7.2 Randomized trials of endovascular treatment stratified by hemodynamic biomarkers

Balloon angioplasty and stenting should be evaluated in separate randomized comparisons. BASIS provides overall randomized evidence for submaximal balloon angioplasty, but its prespecified hypoperfusion analysis did not demonstrate treatment-effect modification (16). A future balloon-angioplasty trial should use a locked multilevel definition of lesion function, distal perfusion, and compensation, with a prespecified treatment × biomarker interaction. ICAS-MT evaluates stenting in an Angio-FF-enriched population, where device-specific procedural risks must be assessed separately.

### 7.3 Randomized trials of blood-pressure Management in low-Flow States

Blood-pressure-dependent low-flow biomarkers may be particularly suitable for evaluation in small, early-phase mechanistic safety trials. After patients have entered a clinically stable phase and progressive stroke has been excluded, biomarker-positive patients could be randomized to either standard guideline-based management or a prespecified, individualized strategy of gradual blood-pressure reduction. Neurological events should be closely monitored, with repeat assessment of perfusion or regional blood flow at predefined time points.

Because VERiTAS included patients with both intracranial and extracranial vertebrobasilar stenosis or occlusion, its observational findings should be regarded primarily as a physiological prior and cannot be directly extrapolated to support relaxation of blood-pressure targets in sICAS (71–73). Accordingly, current guideline-recommended blood-pressure targets should not be modified on the basis of these findings before dedicated randomized evidence becomes available.

### 7.4 Perforator-related risk and evaluation of net clinical benefit

An interventional prediction model should account not only for the potential long-term ischemic benefit of treatment but also for peri-procedural harm. In the randomized trials described above, a perforator-risk stratum could be prespecified, with treatment interactions assessed separately for 30-day perforator stroke and for the overall net clinical outcome. If randomization to intervention is considered ethically unacceptable in an extremely high-risk stratum, evidence from that population would necessarily rely on prospective registries and causal-inference methods. Such evidence may provide important mechanistic and clinical clues but should not be regarded as equivalent to randomized evidence. The 2025 trial excluding adjacent branch disease further suggests that a single exclusion criterion is insufficient for identifying patients with a favorable interventional risk–benefit profile (68). A more comprehensive net-benefit framework should therefore incorporate perforator morphology, lesion location, procedural technique, and the degree of distal hemodynamic decompensation simultaneously (74–79).

### 7.5 Registered studies related to sICAS Hemodynamics and endovascular treatment

CASSISS-DPR and CASSISS-MPR are prospective observational cohorts designed to evaluate risk using DSA-derived DPR and MRI-derived MPR, respectively; they may strengthen prognostic evidence but cannot establish treatment-effect prediction (80, 81).

CRTICAS-2 is a prospective real-world observational cohort of patients undergoing endovascular treatment and may provide information on contemporary procedural safety and allow exploratory evaluation of imaging-related factors; however, confounding in treatment selection remains an important limitation. BAMUS-ICAS is a randomized trial of balloon angioplasty in patients with acute minor unstable stroke, whereas BLAST is registered as a prospective observational cohort. Both studies primarily evaluate early balloon-based treatment strategies rather than prespecified modification of treatment effects by hemodynamic biomarkers (82–84) (Table 5).

**Table 5:** Major relevant registered studies as of September 17, 2026 and the questions they can address.

Study/Status	Design and sample size	Core question	Evidence role it can support	Cannot directly answer
ICAS-MT; NCT05974033; not yet recruiting. Registry status last verified Aug 2023; author check Sep 17, 2026 (70)	Hybrid prospective design; planned *n* = 536: randomized interventional stratum *n* = 336 (Angio-FF ≤ 0.70) and registry stratum *n* = 200 (Angio-FF > 0.70).	Stenting + medical therapy vs. medical therapy in a functionally enriched population.	Treatment main effect within the positive stratum; implementability of the threshold.	Full-range treatment × Angio-FF interaction; stenting effect in the >0.70 stratum.
CASSISS-DPR; NCT07309705; active, not recruiting. Registry status last verified Dec 2025; author check Sep 17, 2026 (80)	Prospective observational cohort; planned *n* = 400.	DSA-derived DPR and 1-year same-territory event risk.	Measurement and prognostic validation.	Relative effect of any treatment.
CASSISS-MPR; NCT07309718; recruiting. Registry status last verified Dec 2025; author check Sep 17, 2026 (81)	Prospective observational cohort; planned *n* = 400.	MRI-derived MPR and stroke risk.	Prognostic validation of a noninvasive functional biomarker.	Treatment interaction among balloon angioplasty, stenting, and medical therapy.
CRTICAS-2; NCT06740513; not yet recruiting. Registry status last verified Dec 2024; author check Sep 17, 2026 (82)	Prospective real-world observational cohort; planned *n* = 1,000.	Efficacy, safety, and multimodal assessment in contemporary interventional practice.	Real-world prognosis, technical safety, and hypothesis generation.	Causal efficacy and predictive interaction without randomized treatment comparison.
BAMUS-ICAS; NCT06763458; recruiting. Registry status last verified Apr 2026; author check Sep 17, 2026 (83)	Randomized, blinded endpoint assessment; planned *n* = 100.	Submaximal balloon angioplasty + medical therapy vs. medical therapy in acute minor unstable stroke.	Overall treatment effect in a specific acute setting.	Predictive evidence if biomarker interaction is not prespecified and tested.
BLAST; NCT06014723; recruiting. Registry status last verified Jan 2024; author check Sep 17, 2026 (84)	Prospective observational cohort; planned *n* = 416.	Early submaximal balloon angioplasty + medical therapy vs. medical therapy in minor stroke/TIA.	Comparative-effectiveness and safety signals.	Randomized causal treatment effect because treatment allocation is nonrandom; treatment × biomarker interaction.

ICAS-MT, IntraCranial Angiography-derived Fraction Flow-guided Percutaneous Transluminal Angioplasty and Stenting Versus Medical Therapy; Angio-FF, angiography-derived fractional flow; CASSISS, China Angioplasty and Stenting for Symptomatic Intracranial Severe Stenosis; DPR, digital subtraction angiography-derived pressure ratio; MPR, magnetic resonance imaging-derived pressure ratio; DSA, digital subtraction angiography; MRI, magnetic resonance imaging; TIA, transient ischemic attack; CRTICAS-2, The New Clinical Registration Trial of Interventional Treatment for Patients With Symptomatic Intracranial Artery Stenosis; BAMUS-ICAS, Balloon Angioplasty for Minor Unstable Stroke With Intracranial Atherosclerosis Stenosis; BLAST, Balloon Angioplasty for Intracranial Atherosclerotic Minor Stroke/Transient Ischemic Attack; NCT, National Clinical Trial number.

### 7.6 Statistical analysis and external validation requirements

The minimum design requirements for predictive-biomarker studies are shown in Table 6.

**Table 6:** Minimum design requirements for predictive-biomarker studies.

Evaluation step or study design	Must be prespecified	Main failure mode	Acceptable evidence
Measurement	Time window, blood pressure/PaCO_2_, acquisition protocol, segmentation and boundary conditions, failure criteria, blinded core laboratory.	Platform differences and physiologic fluctuations are mistaken for true biological differences.	Reproducibility, cross-center consistency, and error propagation meet prespecified standards.
Definition	Biomarker definition (single measure or combination), functional form, and any cutoffs; combination rules where applicable; missing-data handling; and handling of perforator-related risk when relevant to the treatment comparison.	Thresholds and combinations are repeatedly adjusted according to outcomes.	The biomarker definition and analysis rules are locked before independent validation and can be applied reproducibly without modification based on validation outcomes.
Biomarker-enriched randomized trial (if used)	Explicit positive threshold, treatment A vs. B within the positive stratum, management of the negative stratum, and net clinical endpoint.	The treatment main effect within the positive stratum is described as a proven interaction.	Shows that treatment is effective in the prespecified positive population; conclusions remain limited to that population.
Interaction validation	Randomized treatment comparisons across the biomarker range of interest (including both strata if dichotomized); prespecified functional form, primary treatment × biomarker interaction, effect scale, follow-up, and multiplicity control.		The prespecified interaction is reported with effect estimates and confidence intervals; absolute and relative treatment effects are presented across biomarker values, with interpretation based on clinical relevance and precision.
External validation	Independent population, different centers and devices; calibration and implementability; prespecified replication criteria.	Only cross-validation in the same cohort or post hoc subgroup replication is performed.	Treatment-effect patterns and magnitudes are independently replicated across the intended biomarker range. Where prediction models are used, their calibration and ability to predict treatment benefit are evaluated; performance near decision thresholds is assessed when such thresholds are proposed.
Clinical utility	Compare a biomarker-guided strategy with usual decision making; simultaneously assess procedural harm, hemorrhage, fairness, and cost.	Improvement in an imaging measure is treated as patient benefit.	The biomarker-guided strategy improves patient-important outcomes without unacceptable harm.

PaCO_2_, arterial partial pressure of carbon dioxide.

## 8 Standardization of computational models and translation to digital twins

Patient-specific CFD, a one-time risk-prediction model, and a digital twin should not be regarded as synonymous concepts. In the near term, engineering priorities should focus on clearly defining the intended context of use, locking model inputs, algorithms, and quality-control procedures, completing software and computational verification, quantifying uncertainties related to boundary conditions and segmentation, and ensuring that model-credibility requirements are commensurate with the clinical consequences of an incorrect decision (85). In this review, we reserve the term “digital twin” for systems in which a clearly defined closed loop exists between the real patient and the virtual model, incorporating dynamic updating, counterfactual simulation, clinical feedback, and appropriate governance (86, 87). Until predictive validation has been completed, digital-twin approaches should be regarded primarily as a long-term platform for integration and hypothesis testing. They cannot replace randomized treatment comparisons and should not constitute the central claim of this review.

## 9 Discussion

Evidence accumulated over the past decade supports different hemodynamic and imaging measures to varying degrees. Borderzone infarction and distal hypoperfusion are supported by relatively strong prognostic evidence from multicenter cohorts or pooled analyses. Measures of lesion function, including PR/FF, as well as regional arterial flow assessed by QMRA and collateral or cerebrovascular-reserve measures, have also been associated with recurrent risk, although the supporting evidence relies more heavily on single-center studies, platform-specific definitions, or *post hoc* thresholds. A more precise physiological formulation is that lesion function influences tissue perfusion through the combined effects of antegrade residual flow, collateral circulation, and cerebrovascular autoregulation. Compensatory mechanisms may therefore produce discordance between resting CBF and measures such as PR, Tmax, or cerebrovascular reserve (CVR). Internal borderzone infarction is more closely related to the downstream consequences of supply decompensation, whereas embolic and perforator mechanisms may occur in parallel with, or overlap with, hypoperfusion (30–36, 42, 43, 88). The D_2_H_2_A model combines conventional clinical risk factors with CFD-derived hemodynamic status and demonstrates the feasibility of multivariable risk modeling. However, its internally derived C statistic of 0.73 demonstrates discrimination within the development cohort but does not establish external validity, clinical utility, or treatment benefit. Venous outflow should currently remain an exploratory terminal readout.

Among the completed direct sICAS studies identified through searches last updated on September 17, 2026, we found no hemodynamic biomarker with both prospective validation and independent external replication as a treatment-effect modifier for a specific therapy. Angio-FF has been incorporated into the registered ICAS-MT design and therefore has relatively advanced trial-implementation maturity, representing an important step toward treatment-specific validation. However, this does not mean that its biological validity, predictive value, or clinical-translation maturity has already been established. If future results from the randomized Angio-FF ≤ 0.70 stratum demonstrate an acceptable net clinical benefit of stenting plus aggressive medical management, this threshold could become a candidate treatment-selection criterion supported by randomized evidence within the trial-eligible population. Nevertheless, because patients with Angio-FF > 0.70 are not randomized, such a result alone would still be insufficient to establish a formal treatment × Angio-FF interaction or to exclude treatment benefit in the biomarker-negative stratum.

A reasonable near-term research strategy is therefore to use ICAS-MT to complete functionally enriched validation of the stenting pathway while simultaneously measuring lesion function, collateral status or reserve, and tissue perfusion within the same time window and prespecifying the interpretation of cross-level concordant and discordant patterns. Biomarker-stratified randomized trials should then formally test interactions between balloon angioplasty or stenting and a multilevel combination of impaired lesion function, distal tissue hypoperfusion or regional low flow, and failed compensation. In parallel, plaque-related embolic features and perforator-risk profiles should be evaluated as nonhemodynamic harm modifiers of procedure-related injury. Prognostic risk may help define treatment thresholds and estimate absolute benefit, but identifying who is at high risk is not equivalent to identifying which treatment is most appropriate. For clinical implementation, biomarker-guided treatment selection should be supported by reproducible measurements, a clearly specified context of use, reliable evidence on comparative treatment effects and harms, external validation, and demonstrated clinical utility. Claims of treatment-effect modification additionally require supporting interaction evidence on a prespecified effect scale and independent replication.
